# Comparison of Pre-dilution and Post-dilution Methods on Cytokine Clearance Using Polymethylmethacrylate (PMMA) Membrane Hemofilters in Continuous Hemodiafiltration

**DOI:** 10.7759/cureus.77500

**Published:** 2025-01-15

**Authors:** Mototsugu Kudo, Shinya Chihara, Hiroomi Tatsumi, Satoshi Kazuma, Yoshiki Masuda

**Affiliations:** 1 Department of Intensive Care Medicine, Sapporo Medical University School of Medicine, Sapporo, JPN; 2 Department of Clinical Engineering, Japan Healthcare University, Sapporo, JPN; 3 Department of Acute and Critical Care Medicine, Sapporo Higashi Tokushukai Hospital, Sapporo, JPN

**Keywords:** acute kidney injury, adsorption, chdf, cytokine, il-6, pmma, sepsis

## Abstract

In Japan, the post-dilution method is preferred to maintain the dialysis efficiency of low molecular weight substances in continuous hemodiafiltration (CHDF). In contrast, the pre-dilution method is preferable to ensure a stable filter lifetime, especially for membranes with cytokine absorptive characteristics that are at high risk of membrane coagulation. However, the impact of the dilution method on the removal performance of adsorptive membranes for cytokines remains unclear. This study aimed to investigate the effect of different dilution methods on the adsorption clearance of cytokine on polymethylmethacrylate (PMMA) membranes by in vitro experiments. Bovine albumin, interleukin (IL)-6, and creatinine were added to phosphate-buffered saline to achieve final concentrations of 3.5 g/dL, 1000 pg/mL, and 10 mg/dL, respectively. The test solution was passed through the 1.8 m^2^ PMMA membrane hemofilter at a flow rate of 100 mL/min in a single-pass system. The dialysate/filtration flow rates used were 2.5/2.5, 5.0/5.0, and 7.5/7.5 mL/min for each pre- and post-dilution method. Samples were collected seven times from pre- and post-hemofilter every minute, 10 min after the start of CHDF. Samples were frozen and stored, and the concentration of IL-6 was measured by enzyme-linked immunosorbent assay (ELISA), and clearance was calculated. Creatinine clearance increased with an increasing flow rate in pre- and post-dilution, while IL-6 clearance was less affected by flow rate. IL-6 clearance was 35.5 mL/min (32.1-39.6) for pre-dilution and 37.1 mL/min (33.8-41.1) for post-dilution in the 2.5/2.5 setting, and 34.2 mL/min (28.8-36.2) for pre-dilution and 32.4 mL/min (32.4-37.4) for post-dilution in the 5.0/5.0 setting. The dilution methods showed no significant differences. Moreover, in the 7.5/7.5 setting, clearance was 29.5 mL/min (27.7-37.2) for pre-dilution and 39.9 mL/min (37.8-41.5) for post-dilution (P<0.001); however, all clearance values were higher than theoretical values expected from filtration removal. In conclusion, the cytokine removal characteristics of the PMMA membrane hemofilter were not largely affected by the dilution method, and IL-6 clearance could be fully demonstrated even in the pre-dilution method.

## Introduction

Sepsis is known to cause a systemic inflammatory response [1], and if the condition worsens, it can lead to blood perfusion failure in systemic tissues, resulting in multi-organ failure. Notably, 40-50% of acute kidney injuries (AKI) occurring in intensive care units are septic in origin [2]. Additionally, various inflammatory mediators are believed to be critical in septic AKI pathogenesis.

Continuous kidney replacement therapy (CKRT) is commonly used for AKI treatment in emergency and intensive care settings due to the patient’s unstable hemodynamic status. Although CKRT for AKI primarily targets uremic substances such as blood urea nitrogen and creatinine, inflammatory mediators like cytokines are also targeted for removal in septic AKI. There are two methods for cytokine removal in CKRT: using a highly permeable hemofilter such as polysulfone or cellulose acetate to increase the filtration volume or using a cytokine adsorbing hemofilter (CAH). In the case of using a high-permeability hemofilter, increasing the filtration volume in anticipation of therapeutic effects leads to increased medical costs. In contrast, when using CAH such as polyethylenimine-coated polyacrylonitrile (AN69ST) or polymethyl methacrylate (PMMA), the mechanism of removal differs, so cytokine clearance may not depend on the filtration volume. There are particularly many reports on CKRT using PMMA [3-5], suggesting that its adsorption effects may help maintain therapeutic efficacy in removing inflammatory mediators even with low filtration volume settings. PMMA’s homogeneous hydrophobic structure allows for the adsorption and removal of large molecular weight cytokines through a combination of “surface adsorption,” “occlusion of pores,” and “filtration” mechanisms [6].

Also in Japan, post-dilution methods are often used to enhance the removal of low molecular weight substances by filtration dialysis in CKRT. However, post-dilution methods raise concerns regarding filter lifetime reduction due to blood concentration during filtration and membrane clogging. Particularly, in continuous venovenous hemodiafiltration using adsorptive membrane filters, significant mechanical stress on the membrane from adsorption and blood coagulation portends a shorter filter lifetime [7,8]. Membrane clogging requires interruption of treatment for replacement, leading to various impacts such as decreased therapeutic efficacy, increased risk of complications, and rising healthcare costs. The pre-dilution method reduces mechanical stress because the blood is diluted before entering the filter. However, this approach may also decrease the clearance of small molecules [9]. Unlike the removal of small molecules by filtration and dialysis, cytokine removal by PMMA membranes depends on adsorption function. Thus, high clearance may be maintained even with pre-dilution. However, the relationship between the cytokine removal performance of PMMA membranes, the setting conditions in the low convection volume range, and the dilution method has not been thoroughly examined.

Therefore, we performed in vitro experiments using cytokines-containing test solutions. This evaluation aimed to determine the effect of different dilution methods on cytokine removal by PMMA membrane filters.

## Technical report

Materials and methods

Experimental Procedure

**Figure 1:**
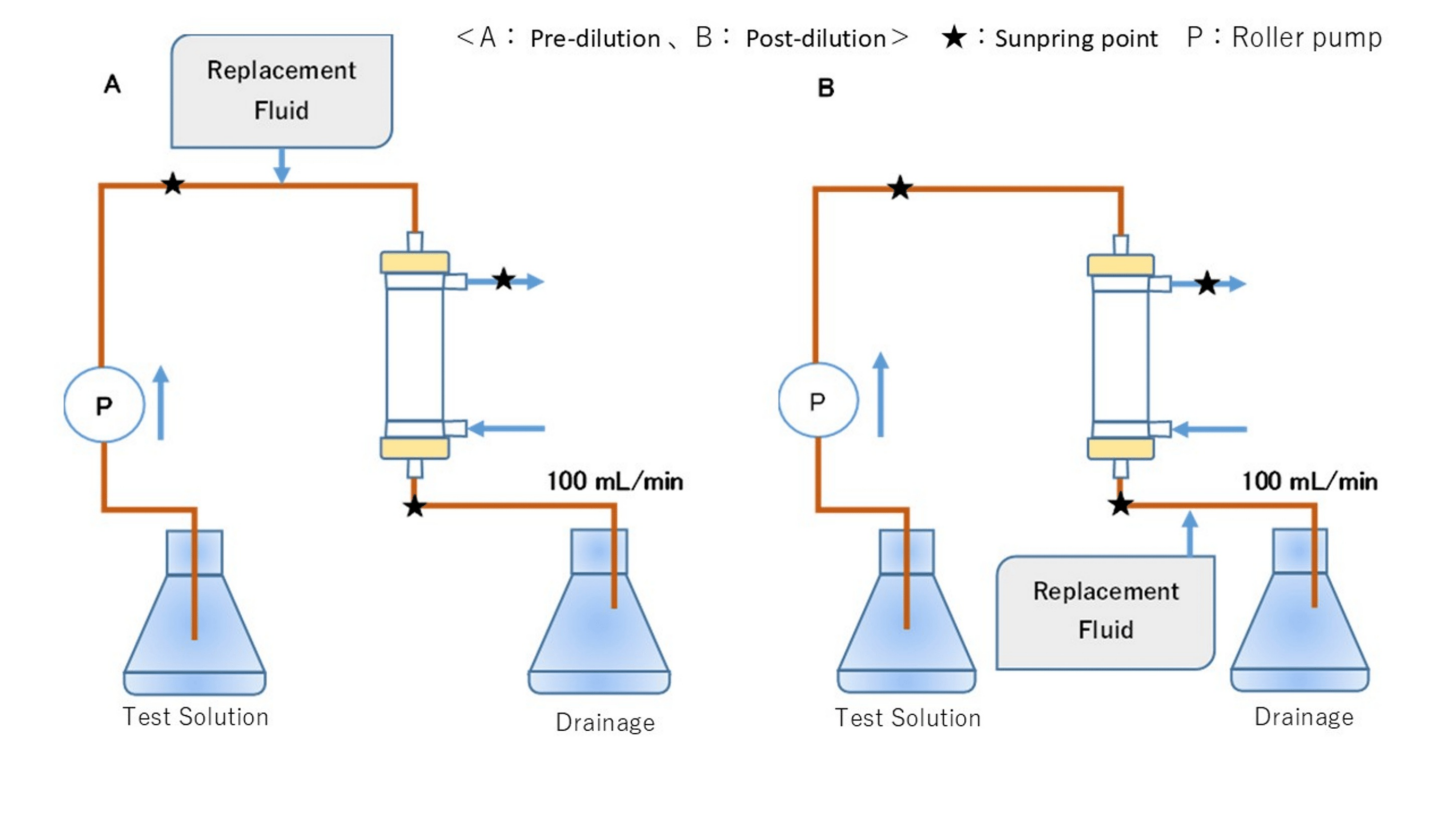
Experimental circuit diagram A: pre-dilution. B: post-dilution. Stars are indicated as sampling points. The circuit, hemofilter, test solution bottle, and waste solution bottle were arranged so that the test solution circulated from the inlet circuit and drained into the outlet circuit in a single-pass system. Circulation was initiated with QD 2.5 mL/min, QF 2.5 mL/min, and test solution flow rate (QT) set at 100 mL/min for circuits A and B, respectively. A total of seven test fluid samples were taken from the inlet and outlet of the hemofilter every minute starting at a steady state 10 minutes after the start of circulation. The same experiment was then performed by changing the QD /QF to 5.0 mL/min each. The same experiment was performed by changing the QD /QF to 7.5 mL/min. This figure was created by the author.

The circuit, hemofilter, test solution bottle, and waste solution bottle were arranged so that the test solution circulated from the inlet circuit and drained into the outlet circuit in a single-pass system. Figure 1 depicts the experimental circuit diagram. The hemofilter’s membrane material was PMMA (HEMOFEEL CH-1.8W®, Toray Medical Co., Ltd., Tokyo, Japan). The circuit used in the experiments, the hemofilter, was replaced with a new one each time the dialysis flow rate (QD) and filtration flow rate (QF) settings and dilution method were changed.

For the experimental conditions, QD 5.0 mL/min and QF 5.0 mL/min, which are commonly used purification volume settings in CKRT with PMMA, were used as intermediate settings (medium settings), while QD 2.5 mL/min and QF 2.5 mL/min (low settings), and QD 7.5 mL/min and QF 7.5 mL/min (high settings) were used as three additional QD/QF settings. We compared creatinine and IL-6 clearance using different dilution methods (pre-dilution and post-dilution methods) for each QD/QF setting. The test solution flow rate (QT) was 100 mL/min.

The test solution was collected from the inlet and outlet of the hemofilter from the steady state established 10 min after the start of the experiment. The collection of the test solution was repeated seven times every minute. The collected samples were stored at -80℃. Creatinine levels were measured using the enzymatic method, while IL-6 levels were assessed using a commercially available enzyme-linked immunosorbent assay (ELISA) kit following the manufacturer’s instructions. The clearance of each solute was determined using the following equation [10]: for the pre-dilution method, CKRT clearance (mL/min) = (Cti - Cto) / Cti × QT, and for the post-dilution method, CKRT clearance (mL/min) = (Cti - Cto) / Cti × (QT - QF) + QF, where Cti and Cto represent the concentrations of substances at the inlet and outlet of the hemofilter, respectively.

Preparation of Solution

To achieve a final concentration of 3.5%, we added 630 mg of bovine albumin (Nacalai Tesque, Kyoto, Japan) to 18 L of 10% phosphate-buffered saline solution (GIBCO®, Life Technologies, Massachusetts, USA). Additionally, we added 1,800 mg of creatinine (Nacalai Tesque, Kyoto, Japan) and 18 µg of interleukin (IL)-6 (Sigma-Aldrich, St. Louis, USA). The solute concentrations were creatinine at 10 mg/dL and IL-6 at 1,000 pg/mL. The circulating solution was heated to 37.0℃ and continuously stirred using a magnetic stirrer.

Statistical Analysis

Continuous variables were expressed as medians (interquartile range). The Friedman test was used to test for three or more groups, and the level of statistical significance was set at a P<0.05 as the post-hoc test. Statistical analysis was performed using EZR.

Results

Creatinine Clearance

**Figure 2:**
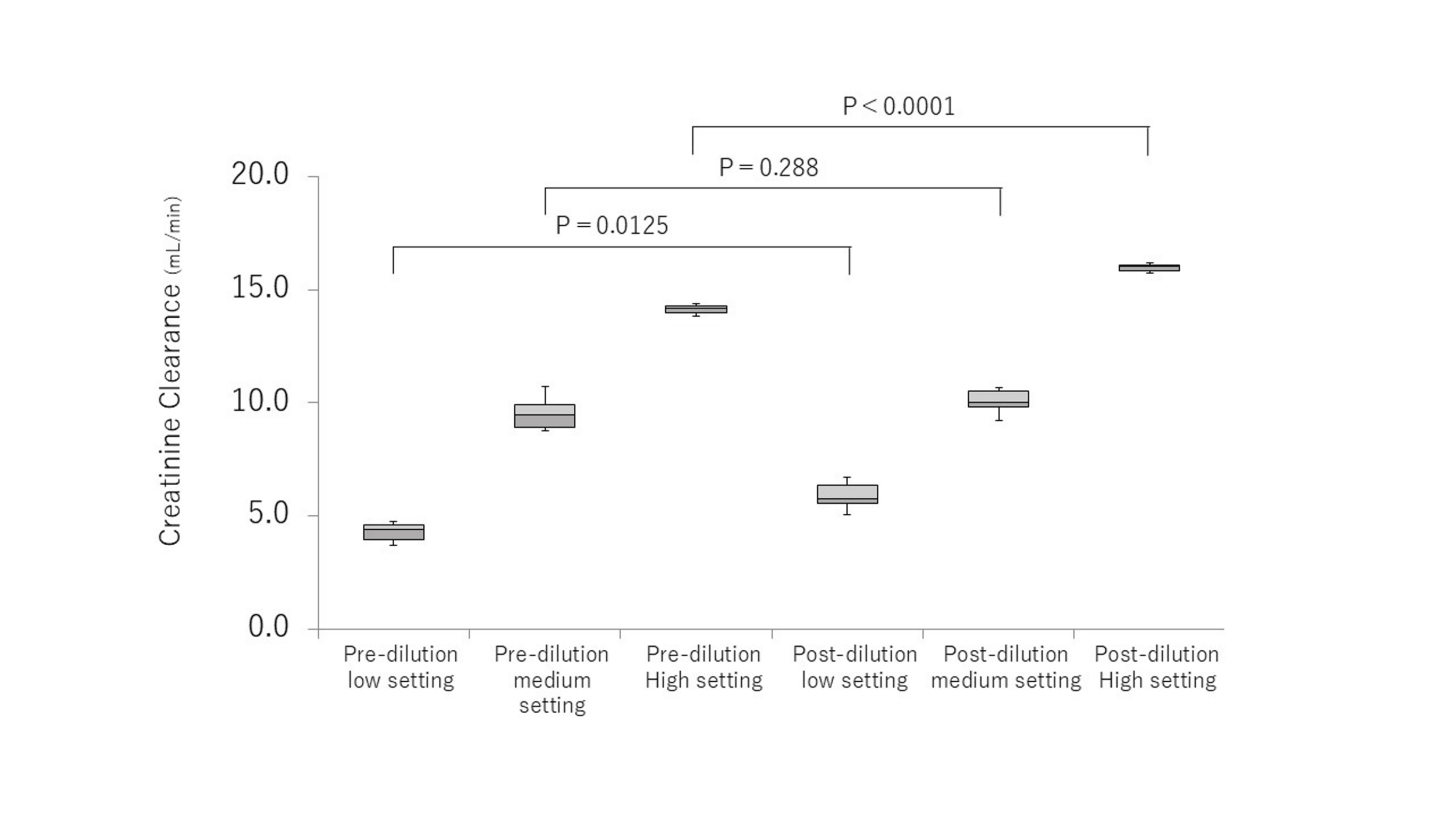
Creatinine clearance of CHDF Creatinin clearance was compared between pre-dilution and post-dilution under low (QF/QD = 2.5/2.5), medium (5.0/5.0), and high (7.5/7.5) settings, respectively. Box plot, center lines indicate the medians; box limits indicate the 25th and 75th percentiles; whiskers extend to minimum and maximum values. CHDF, continuous hemodiafiltration This figure was created by the author.

Creatinine clearance was compared using different QD/QF settings (Figure 2). Clearance increased as QD/QF increased for the post-dilution and pre-dilution methods (P<0.001). In the low setting, clearance was 4.4 mL/min (4.0-4.6) and 5.8 mL/min (5.5-6.4) for pre-dilution and post-dilution, respectively, with significantly higher clearance in the post-dilution (P=0.012). Additionally, clearance in the high setting was also significantly different: 14.2 mL/min (14.0-14.3) for pre-dilution and 16.0 mL/min (15.8-16.1) for post-dilution (P<0.001). However, clearance in the medium setting showed no significant difference between the dilution methods at equivalent purification volumes: 9.5 mL/min (8.9-9.9) for pre-dilution and 10.0 mL/min (9.8-10.5) for post-dilution.

IL-6 Clearance

**Figure 3:**
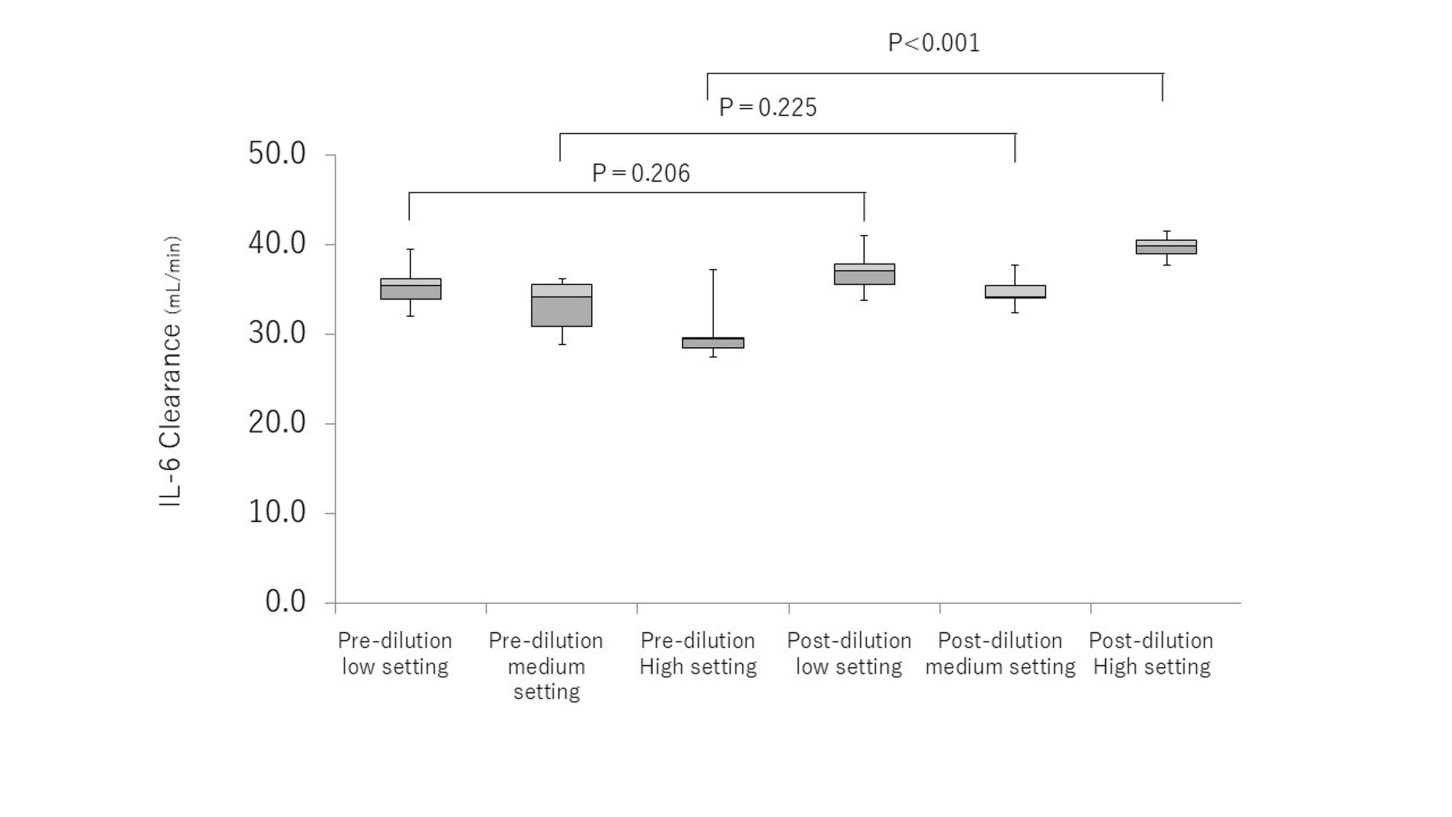
IL-6 clearance of CHDF IL-6 clearance was compared between pre-dilution and post-dilution under low (QF/QD = 2.5/2.5), medium (5.0/5.0), and high (7.5/7.5) settings, respectively. Box plot, center lines indicate the medians; box limits indicate the 25th and 75th percentiles; whiskers extend to minimum and maximum values. CHDF, continuous hemodiafiltration This figure was created by the author.

In the low setting, the IL-6 clearance was 35.5 mL/min (34.0-36.2) and 37.1 mL/min (35.6-37.8) for pre-dilution and post-dilution, respectively, with no significant difference (P=0.206) (Figure 3). Similarly, in the medium setting, the IL-6 clearance was 34.2 mL/min (30.9-35.6) and 34.2 mL/min (34.1-35.5) for pre-dilution and post-dilution, respectively, with no significant difference (P=0.225). In contrast, in the high setting, the clearance was 29.5 mL/min (28.5-29.6) and 39.9 mL/min (39.0-40.5) for pre-dilution and post-dilution, respectively, with the post-dilution clearance being significantly higher (P<0.001).

## Discussion

When continuous hemodiafiltration (CHDF) is performed for septic AKI, large molecular weight mediators such as inflammatory cytokines are also targeted for removal. CHDF using PMMA membrane can efficiently remove cytokines by adsorption to the membrane. However, PMMA membranes have lower permeability than polysulfone and other highly permeable membranes. Therefore, fouling [11] and platelet activation-induced membrane coagulation [12] are concerns for CHDF using PMMA membranes. CHDF with pre-dilution can reduce mechanical stress on the membrane; however, it is thought to decrease solute clearance. Although the relationship between solute clearance and dilution method in continuous hemofiltration using polysulfone membrane has been studied previously, the relationship between large-molecule mediators and dilution method in CHDF using PMMA membrane has not been fully investigated. In this study, we performed in vitro experiments using test solutions containing cytokines to investigate the effect of different dilution methods on cytokine clearance of PMMA membranes.

As a result, IL-6 clearance was higher than the theoretical value in all QD/QF settings, unaffected by the dilution method, due to the adsorption effect of the membrane. Therefore, we believe that the combination of PMMA membrane and pre-dilution can be used to maintain cytokine clearance and prolong filter lifetime in CHDF for septic AKI [13], in which cytokine removal is an important objective. If the frequency of circuit replacement decreases due to extended filter life, it could not only enhance therapeutic efficacy but also reduce the risk of complications associated with circuit replacement. Moreover, the higher-than-expected IL-6 clearance, regardless of the dilution method or convection volume, suggests that pre-diluted CHDF with a smaller convection volume may be highly effective in adsorbing cytokines, highlighting the potential cost-effectiveness of PMMA-CHDF.

In this study, QD and QF were used at 2.5 mL/min, 5.0 mL/min, and 7.5 mL/min settings for two reasons. First, Japanese medical insurance claims limit the amount of dialysate and replacement fluid used. Second, the PMMA membrane has low permeability, and the ratio of QT to QF limited the QF setting in this experiment. However, even at such low QD and QF, IL-6 clearance was higher than those in previous reports using other membranes.

Since the QD/QF of CHDF was significantly lower than the blood flow rate (QB), clearance was dependent on the QD/QF. The theoretical upper limit of clearance in CHDF is QD/QF. In CHDF with filtration, the post-dilution method was thought to have a higher solute clearance than the pre-dilution method due to the dilution effect. However, IL-6 clearance between the low and medium settings of the volume of QD and QF was not different. Since the adsorption mechanism of PMMA membrane is the occlusion of pores that occurs with filtration, the increased flow rate at the inlet of the hemofilter due to the pre-dilution method could have promoted internal filtration, resulting in an enhanced adsorption effect regardless of the QD/QF settings or dilution method. Therefore, the adsorption efficiency of large-molecule mediators in CHDF with PMMA membrane in the low QD/QF settings, which was the experimental condition used in this study, did not necessarily depend on the dilution method. If the adsorption effect is enhanced by promoting internal filtration, increasing QB could be expected to further improve the therapeutic effect. Additionally, increasing QB would help prevent blood retention, which could be even more effective in reducing the risk of membrane coagulation.

In the high setting, the IL-6 clearance of the pre-dilution method was 29.5 mL/min (28.5-29.6), which was significantly lower than that of the post-dilution method. However, in Japan, the daily volume of replacement fluid is limited to approximately 10 mL/min for both dialysis and filtration flow for medical insurance claims. Therefore, the high setting exceeded the upper limit of the medical insurance claim, and the medium setting was the upper limit of the setting. Furthermore, considering that the mechanical stress on the membrane increases with the QD/QF increase in the post-dilution method, the pre-dilution method may have an advantage in prolonging the filter lifetime even in the high setting.

PMMA-CHDF for septic AKI requires the removal of low molecular weight substances such as uremic substances, so the post-dilution method has been widely used. The study showed an advantage of the post-dilution method, especially in clearance in the high setting, but the difference was small. However, various disadvantages of the post-dilution method in CHDF compared to the pre-dilution method have been reported. For example, it has been reported that cytokine clearance decreases with time in the post-dilution method compared to the pre-dilution method due to membrane fouling [14]. Similarly, membrane fouling is thought to have various effects, including a decrease in filter lifetime and an increase in medical costs and personnel burden [13]. In addition, it has been reported that the post-dilution method promotes an excessive immune response of blood cells compared to the pre-dilution method [15]. Such biocompatibility effects may also be relevant to therapeutic efficacy, especially since the conditions targeted by PMMA-CHDF include septic AKI. Therefore, the pre-dilution method is superior to the post-dilution method for PMMA-CHDF in terms of filter lifetime and biocompatibility.

The following limitations exist in this study. First, this study was conducted in vitro using a test solution. In actual clinical practice, substances such as blood cells and proteins present in blood are known to reduce the efficiency of filtration and adsorption. Clinical studies under similar conditions are necessary to support the results of the present study. The only cytokine added to the test solution was IL-6. In the future, it will be necessary to examine the differences in adsorption effects for each substance using test solutions that include a variety of substances involved in sepsis, in addition to IL-6. Second, this study was performed only on PMMA membranes and could not be compared with other membranes.

Furthermore, the number of mediators added to the test solutions was limited. Future comparisons with other membranes and comparisons with a wider range of mediators are needed.

## Conclusions

The differences in cytokine clearance based on the dilution method of PMMA-CHDF were investigated in vitro. PMMA-CHDF showed no difference in IL-6 clearance by dilution method in the range below 5.0 mL/min, a common setting for QD and QF. In CHDF using PMMA membrane hemofilter, pre-dilution was an effective method for cytokine adsorption and filter lifetime.
